# Biochemical and phenological characterization of diverse wheats and their association with drought tolerance genes

**DOI:** 10.1186/s12870-023-04278-9

**Published:** 2023-06-19

**Authors:** Iftikhar Ali, Saeed Anwar, Ahmad Ali, Zahid Ullah, Dalal Nasser Binjawhar, Hassan Sher, Usama K. Abdel-Hameed, Muhammad Aamir Khan, Khawar Majeed, Mariusz Jaremko

**Affiliations:** 1 Center for Plant Science and Biodiversity, University of Swat, Charbagh Swat, 19120 Pakistan; 2 Department of Genetics and Development, Columbia University Irving Medical Center, New York, NY 10032 USA; 3 School of Life Sciences & Center of Novel Biomaterials, The Chinese University of Hong Kong, Hong Kong, 999077 Hong Kong; 4 Department of Chemistry, College of Science, Princess Nourah Bint Abdulrahman University, Riyadh, 11671 Saudi Arabia; 5 Biology Department, College of Science, Taibah University, Al-Madinah Al-Munawarah, 42353 Saudi Arabia; 6 Botany Department, Faculty of Science, Ain Shams University, Cairo, 11566 Egypt; 7 Department of Plant Sciences, Quaid-I-Azam University, Islamabad, Pakistan; 8 Smart-Health Initiative (SHI) and Red Sea Research Center (RSRC), Division of Biological and Environmental Sciences and Engineering (BESE), King Abdullah University of Science and Technology (KAUST), Thuwal, 329555-6900 Saudi Arabia

**Keywords:** Wheat, Drought, Phenological, Biochemical, Functional Marker, KASP

## Abstract

Drought is one of the most important wheat production limiting factor, and can lead to severe yield losses. This study was designed to examine the effect of drought stress on wheat physiology and morphology under three different field capacities (FC) viz. 80% (control), 50% (moderate) and 30% (severe drought stress) in a diverse collection of wheat germplasm including cultivars, landraces, synthetic hexaploid and their derivatives. Traits like grain weight, thousand grain weight and biomass were reduced by 38.23%, 18.91% and 26.47% respectively at 30% FC, whereas the reduction rate for these traits at 50% FC were 19.57%, 8.88% and 18.68%. In principal component analysis (PCA), the first two components PC1 and PC2 accounted for 58.63% of the total variation and separated the cultivars and landraces from synthetic-based germplasm. Landraces showed wide range of phenotypic variations at 30% FC compared to synthetic-based germplasm and improved cultivars. However, least reduction in grain weight was observed in improved cultivars which indicated the progress in developing drought resilient cultivars. Allelic variations of the drought-related genes including *TaSnRK2.9-5A*, *TaLTPs-11*, *TaLTPs-12*, *TaSAP-7B-*, *TaPPH-13*, *Dreb-B1* and *1fehw3* were significantly associated with the phenological traits under drought stress in all 91 wheats including 40 landraces, 9 varieties, 34 synthetic hexaploids and 8 synthetic derivatives. The favorable haplotypes of *1fehw3*, *Dreb-B1*, *TaLTPs-11* and *TaLTPs-12* increased grain weight, and biomass. Our results iterated the fact that landraces could be promising source to deploy drought adaptability in wheat breeding. The study further identified drought tolerant wheat genetic resources across various backgrounds and identified favourable haplotypes of water-saving genes which should be considered to develop drought tolerant varieties.

**Supplementary Information:**

The online version contains supplementary material available at 10.1186/s12870-023-04278-9.

## Introduction

Bread wheat (*Triticum aestivum* L, Poacea) is an annual grass and important cereal crop. It is the most vital source of nutrition and an important food for nearly 36% of the world’s population [1]. It also provides a significant amount of several components which are beneficial for human food and health such as protein, vitamin B, dietary fibres and phytochemicals The demand for wheat is expected to increase by 50% by the end of 2050 [2–6]. Wheat crops face various biotic and abiotic stresses which affect crops yield globally. Different mechanisms have been adopted by plants to withstand wide range of biotic and abiotic stress [7–11]. Drought stress significantly affects the plant vegetative and reproductive growth [12]. It leads to reduction of stomatal closure, reduction of water content, turgor loss and sometimes it may lead to death of the plants by disturbing metabolism [13]. Further drought stress also influences flowering times in plants [14, 15]. Comparatively drought stress affect protein quality and reduce the grain yield of wheat [16–19]. Drought stress can reduce plant growth and cause a reversible reduction in leaf water content, photosynthetic activity, membrane stability and increase the formation reactive oxygen species (ROS), lipid peroxidation and membrane injury [20, 21].

Improvement of bread wheat to withstand drought and other stresses may be achieved by incorporating genetic variations from wild relatives, which are known to have greater variability for drought tolerance [22, 23]. Landraces and wild species represent a reservoir of favourable alleles for drought tolerance [24, 25]. Landraces are a dynamic population of cultivated plants with historical origin, distinct identity, often genetically diverse and locally adopted, and commonly used by farmer for selection of seeds. Landraces offer a gene pool that enhances biodiversity, and maintains, and stabilizes ecosystems in a viable way to functionalize them [26, 27]. Synthetic hexaploid (SH) wheats were produced earlier from the crosses between tetraploid (*Triticum turgidum* L.) and diploid (*Aegilops tauschii* Coss.) the ancestors of bread wheat [28, 29]. Synthetic hexaploid wheat represents a wide range of genetic variation because of the introduction of supplementary genomic resources from their relatives and acquire enhanced characters like better grain quality, high yield and resistance to various environmental conditions [30, 31]. It has several advantages over common wheat in terms of early maturity, stem diameter, peduncle length, higher thousand grain weight (TGW), dwarfing, harvest index and yield. Other than valuable agronomic and desirable quantitative traits, synthetic wheat has several potentials to improve drought tolerance of common wheat. Synthetic wheat has been reported to have significant genetic variation for drought stress resistance and thus are valuable sources of drought tolerance genes [32–34]. It has proven a good source of tolerance to *Puccinia recondite* [35], *Puccinia striiformis* [36], powdery mildew [37], and, tolerance to water stress [38, 39].

Physiological and biochemical adaptations of wheat landraces to drought stress have pronounced effects on its survival, growth and yield [40]. It is considered important that biochemical attributes need to be considered as superior traits while selecting drought tolerant wheat varieties which can be accomplished by introducing drought tolerant genes to modern wheat [41]. In wheat, several genes having significant contribution in osmotic stress tolerance by producing variety of enzymes and proteins have been reported. Some of these include helicase, proline, Rab (responsive to abscisic acid), rubisco, Lea (late embryogenesis abundant protein) and GST (glutathione-S-transferase) during water deficit stress [42, 43]. It is believed that understanding plant drought stress responses can be achieved by comparing genotypes relative yield in controlled and drought-stress environments. Some researchers believe in selection under drought stress environment, while, others in controlled/normal condition, still, some rely performances under both favorable control and stress conditions [44]. Accordingly, Fischer and Maurer [45] suggested STI (stress susceptibility index) for measurement of yield stability in variable environments. Stress tolerance index (STI) by Fernandez [46], detect genotypes with high yield potential both under control and stressed environments. Similarly, GMP (geometric mean productivity) has its main focus on relative performances.

Marker-assisted selection (MAS) in breeding programs can successfully be accomplished with the help of allele-specific markers [47]. Kompetitive allele specific polymerase chain reaction (KASP) is a simple fluorescence based methodology for indel (insertion/deletion) or SNP (single nucleotide polymorphism) genotyping assays for amplification of DNA samples using a thermal cycler, enabling bi-allelic scoring at a specific locus and hence, offer exceptionally high precision and robustness at a relatively low cost [48]. KASP assays have been successfully designed for different environmental stresses including drought through genetic diversity analysis used for enhancement of wheat [49]. For example, *Dreb* genes have involvement in tolerance to abiotic stresses including low temperature, ABA and drought, while the genetic mapping of *Dreb-B1* may be useful in wheat breeding program for drought tolerance [50]. *TaSnRK2.*9-5A is a drought responsive gene present in wheat which is strongly related to drought stress tolerance, which greatly assists drought tolerance in wheat and shows a beneficial genetic resource for enhancement of drought-tolerant genotype production [51, 52]. *TaLTP* are a drought responsive genes and significantly linked with ideal plant height (PH) under stress environment [53]. Association assessment amongst allelic variation of *TaPPH-7A* and phenological parameters showed that *TaPPH-7A* significantly linked with chlorophyll content, higher grain weight (GW) and thousand grain weight [47, 54]. The exploration of diverse wheat germplasm collection for drought tolerant genes is essential for wheat breeders in order to have knowledge about favorable variations in grain yield attributes as well as to evaluate the influences of selection pressure on promising haplotypes. Previously, Rehman et al*.* [47] tested alleles related to water-deficit tolerance in 153 diverse wheats and concluded that the developed molecular markers tool kit will be helpful for the wheat-breeding programs. However, a comparative assessment between locally adapted landraces, cultivars and advanced lines developed using synthetic hexaploid wheat will provide more insight into identifying promising sources for drought adaptability along with allelic information to select the drought tolerant germplasm. Such comparisons at both genotypic and phenotypic levels have not been performed previously. The objectives of the current study were to investigate the effect of drought stress on the morphological and biochemical attributes of diverse wheats, identify the alleles of genes underpinning major phenotypic variations for drought tolerance and identify the association of those alleles with the phenotypes.

## Methodology

### Experimental germplasm

The experimental germplasm was a collection of diverse wheats which comprised of 40 landraces, 9 varieties, 34 synthetic hexaploids and 8 synthetic derivatives. The landraces were representing different climate zones of Pakistan, and, wheat varieties included both irrigated (drought susceptible) and rainfed (drought tolerant). Similarly, synthetic wheats were previously developed at CIMMYT (International Maize and Wheat Improvement Center) by artificially crossing the elite tetraploid wheats (*Triticum turgidum,* 2n = 2 ×  = 28, AABB) with different accessions of *Aegilops tauschii* (2n = 4 ×  = 14, DD), the F_1_ hybrids (2n = 3 ×  = 21, ABD) were then treated with colchicine which caused chromosome doubling and resulted in fertile synthetic hexaploids, while, synthetic derived wheats were produced by crossing primary synthetics with susceptible bread wheat cultivars [55]. These along with synthetic hexaploids were collected from National Agriculture Research Centre (NARC), Islamabad, and, are available at Centre for Plant Science and Biodiversity, University of Swat, Pakistan. The authors declare that all the permissions or licenses were obtained to collect the wheat plant and that all study complies with relevant institutional, national, and international guidelines and legislation for plant ethics in the methods section. The germplasm details including pedigree is provided in ESM 1.

### Pot experiment and drought stress treatment

A greenhouse experiment was carried out during wheat growing season at Center for Plant Sciences and Biodiversity, University of Swat (34°80’N, 72°35’E). Average relative humidity in the green house was 53%, while, the average day and night temperature was 29 ± 1 °C and 13 ± 2 °C, respectively, during entire duration of the experiment. Viable seeds of the experimental germplasm were washed with 70% ethanol for about 2–3 min, followed by surface sterilization through treating with 20% solution of sodium hypochlorite for 30 min, rinsing with distilled water, and drying with clean tissue paper as previously described in Ali et al., [56]. Growth medium used in the experiment was composed of soil, sand, and clay (2:1:1) which was analysed for its physio-chemical properties. Texture of the experimental soil particles was loamy sand having pH 6.6 with low organic matter (0.69%). The soil particle was slightly calcareous having adequate phosphorus (15 ppm) while low nitrogen (0.034%) and potassium (40 ppm). Ten viable seeds of each genotype were sown in plastic pots with drainage holes filled with the same soil. Germination was carried out under non-stressed condition and after the emergence of seedlings, they were exposed to sun light and normal agriculture practices including thinning and weeding carried out for better growth of the plants. Briefly, thinning was carried out after 2^nd^ and 3rd week of germination, respectively, until 3 uniform sized seedlings per pot were maintained for subsequent studies. Each pot was irrigated with tap water (pH 7.6 and electrical conductivity 1.2 dsm^−1^) to 80% field capacity (FC) till the onset of drought stress treatment. The entire experiment was carried out under a rainout shelter which assisted in eliminating effects of undesired precipitation events and establishing controlled drought stress environment. At pre-anthesis stage drought stress was applied through gravimetric method as discussed previously [57], since, 30% FC in general is considered as severe water stress for crop plants including wheat [58–60]. There were three treatment of drought stress applied as 30% field capacity (severe stress; leaf water potential, LWP = -2.15 MPa), 50% field capacity (moderate stress; LWP = -1.30 MPa) and 80% field capacity (control; LWP = -0.50 MPa). During this study experiment was laid out according to Randomize complete block design (RCBD) for both control and drought stress condition with three times replication. Drought stress was maintained by weighing each pot daily and adding water till reaching their field capacity regimes. Simultaneously, reconciliation of soil moisture in pots was also monitored with the help of TDR soil moisture meter (Spectrum Technologies, Illinois, USA). In order to avoid any positional effect, pots were randomly moved with 2 d interval.

After two weeks of drought stress imposition, till the appearance of visible wilting in plants grown at 30% FC, sampling for subsequent studies was accomplished. Briefly, the uppermost, fully expanded youngest leaves in all tillers of single plant from each pot were harvested, followed by its further splitting as: five leaves were oven dried for 48 h at 65 °C for determination of proline content; seven leaves were dedicated for determination of chlorophyll and relative water contents (RWC); and, four leaves for antioxidant enzyme assays, hence, were instantly put into liquid N and stored at − 45 °C, explained below in the preceding section. Stress was sustained till headings was completed and then typical irrigation was resumed. The experiment was further continued, and data was recorded from remaining intact plants per pot for days to headings, physiological maturity, plant height (cm), spike length (cm), seeds per spike, grain mass and thousand grain weight according to Zadok scale as previously discussed in Yashavanthakumar et al. [61]. The same intact and mature plants were harvested and weighed for biomass and calculating harvest index as previously described by Chowdhury et al. [62] and Afzal et al. [63].

Stress tolerance index (STI) was calculated as described by Ebrahimiyan et al. [44] and Irani et al. [64], according to the following equation;

$$\mathrm{STI}=\frac{{Y}_{p}\times {Y}_{s}}{{\left({\overline{Y} }_{p}\right)}^{2}}$$

Where *Yp* is the cultivar yield potential under drought stress conditions; *Ys* is the cultivar yield potential under non stress conditions; ${\overline{Y} }_{p}$ is the mean yield of all test cultivars under non stress conditions.

### Physiological and biochemical analysis

Relative water content was determined according to Schonfeld et al. [65] by using the following formula.

$$RWC=\frac{Fresh\;weight-Dry\;weight}{Turgid\;weight-dry\;weight}\times100$$

Measurement of chlorophyll content was carried out according to Hiscox and Israelstam, [66] using dimethyl sulphoxide (DMSO). The equations given in Arnon [67] were followed for quantification of chlorophyll contents. Proline content was measured according to the methodology reported in Bates et al., [68]. Superoxide dismutase (SOD) and peroxidase (POD) activities were determined following the methodology reported by Beauchamp and Fridovich [69] and Gorin and Heidema [70].

### Genotyping

For DNA extraction, 5 to 6 cm pieces of leaf tissues were harvested as earlier described in Aboul-Maaty et al. [71]. DNA extraction was carried out using CTAB method with minor modifications if required.

In total eight KASP markers for the genes *TaSnRK2.9-5A, TaLTPs-11, TaSAP-7B, TaPPH-13, Dreb-B1, and 1fehw3* were used*.* (ESM 2). Genotyping was carried out as described previously by Khalid et al. [72] and Rehman et al. [47]. Briefly, a 5 μL of total reaction volume 2.2 μL of 50 ng μL^−1^ DNA sample was dispensed to 384 well microtiter plates and dried in an incubator at 50 °C for 30 min. Then KASP mixture assay containing 2.5 μL KASP (2x) mix, (0.056 μL of allele-specific and common primer) followed by PCR water 2.4 μL and 0.08 μL MgCl_2_ were dispensed to DNA samples. Then the plates were sealed to avoid evaporation of mixture during PCR. At the end of the reaction fluorescence clusters were observed and organized using Kluster Caller software. KASP assay genotyping was carried out in a Real-Time PCR Bio-Rad CFX384TM using Bio-Rad hard shell 384-well PCR Plates.

### Statistical analysis

In the ANOVA model, phenotypic effect was partitioned into overall mean, treatment effect, replication (i.e. block) within treatment effect, genotypic effect, genotype by treatment effect, and random error effect. Let *y*_lij_ be the observed value of a trait of interest for the *i*^th^ accession in the *j*^th^ replication under the *l*^th^ treatment. The linear model used in ANOVA is therefore,

$$ylij = \mu + Dl + Rj/l + Gi + EL + GEil + \varepsilon lij$$

where *l* = 1, 2, …, *L* (*L* = 2 for well-watered and water-limited treatments), *i* = 1, 2, …, *n* (*n* = 91), *j* = 1, 2, …, *r* (*r* = 3), *µ* is overall mean of the whole population, *R*_*j/l*_ is the *j*^th^ replication effect in the *l*^th^ treatment, *G*_i_ is genotypic effect of the *i*^th^ accession, *E*_l_ is treatment effect of the *l*^th^ treatment, *GE*_*li*_ is interaction effect between the *i*^th^ accession and the *l*^th^ treatment, and *ε*_*ljk*_ is random error effect which was assumed to be normally distributed with a mean of zero, and variance ${\upsigma }_{\upvarepsilon }^{2}$. The ANOVA described above was implemented with the GLM procedure in SAS software [73].

All data were organized using Microsoft Excel 365 and JAMOVI (version 1.8; The Jamovi project, 2021) was used for descriptive statistics, coefficient of correlation and principal component analysis (PCA) using correlation method. Student’s T test was used to check the effect of allelic variation of studied traits if only two alleles were identified for any gene. However, a Kruskal–Wallis test was used of number of alleles to be compared are more than two. The R package *ggplot2* was used to draw boxplots for allelic comparisons [74].

## Results

### Phenotypic variation in diversity panel for drought adaptive traits

In this study, 91 wheat accessions including landraces, cultivars, synthetic derivative and synthetic hexaploids were analyzed for important phenological and physio-biochemical traits under three different moisture conditions. Furthermore, the accessions were screened for allelic variations of the genes associated with drought tolerance.

At 50% field capacity (FC), reduction in the agronomic traits ranged between 51.46% to 7.20%. Summary statistics of the genotypes under different levels of drought stress revealed that GW and TGW were reduced by 38.23% and 18.91% at 30% FC, while, it reduced by 19. 57% and 8.88% in 50% FC as compared to control (Table 1). Similarly, RWC decreased to 22.46% and 12.08% at 30% and 50% FC, respectively.

**Table 1:** Summary statistics of the studied phenological and biochemical traits in diverse wheats under three different water treatments including 80, 50 and 30% field capacity

Traits	Mean			Range			CV(%)			% Change in 30% FC	% Change in 50% FC
	Control	30% FC	50% FC	Control	30% FC	50% FC	Control	30% FC	50% FC		
DH (d)	113.58	100.08	106.47	22.00	25.00	23.00	24.55	44.72	29.80	11.89	6.01
PM (d)	146.60	128.70	138.45	38.00	47.00	40.00	152.70	154.73	149.61	12.21	5.56
PH (cm)	86.02	68.28	75.51	37.00	34.00	33.00	57.40	47.23	48.66	20.62	12.21
SPL (cm)	13.08	11.56	11.74	9.50	9.30	7.20	2.50	4.14	1.86	11.66	10.27
SPS	45.52	33.92	39.93	38.00	44.00	43.00	75.05	114.85	88.18	25.48	12.26
GW (g)	1.96	1.21	1.58	3.42	2.56	2.95	0.25	0.26	0.24	38.23	19.57
TGW (g)	42.98	34.86	39.16	54.69	44.31	51.46	39.68	39.67	37.59	18.91	8.88
Biomass (g)	6.34	4.66	5.16	8.57	10.16	8.09	3.01	5.48	3.93	26.47	18.68
HI	0.33	0.28	0.32	0.45	0.52	0.49	0.004	0.01	0.01	13.19	2.15
RWC (%)	17.30	13.41	15.21	20.82	16.97	18.65	15.19	8.72	10.96	22.46	12.08
Proline (µmol/g)	8.64	25.09	12.90	16.67	34.47	24.06	6.65	43.01	14.43	65.56	33.00
Chla (µg/g)	0.28	0.16	0.21	0.20	0.15	0.12	0.002	0.001	0.001	40.36	23.27
Chlb (µg/g)	0.25	0.19	0.21	0.38	0.27	0.37	0.007	0.004	0.005	21.46	14.98
TChl (µg/g)	0.52	0.36	0.42	0.48	0.31	0.46	0.011	0.005	0.462	31.42	19.35
POD (units/g)	5.96	17.19	7.53	18.55	15.00	3.00	4.80	10.79	0.47	65.31	20.76
SOD (units/g)	31.91	46.98	38.05	23.79	19.61	14.47	18.77	16.59	12.86	32.08	16.14

*DH* days to headings, *PM* physiological maturity, *PH* plant height, *SPL* spike length, *SPS* seeds per spike, *GW* grain weight, *TGW* thousand grain weight, *HI* harvest index, *RWC* relative water content, *Chl* chlorophyll, *TChl* total chlorophyll, *POD* peroxidase, *SOD* superoxide dismutase, *FC* field capacity, *CV* coefficient of variation; % Change, percent change

Analysis of variance (ANOVA) revealed that genotypes, treatments, and their interactions were highly significant (*P* < 0.001) for all the studied phenological and physio-biochemical parameters to the applied treatment of drought stress (ESM 3).

**Fig. 1:**
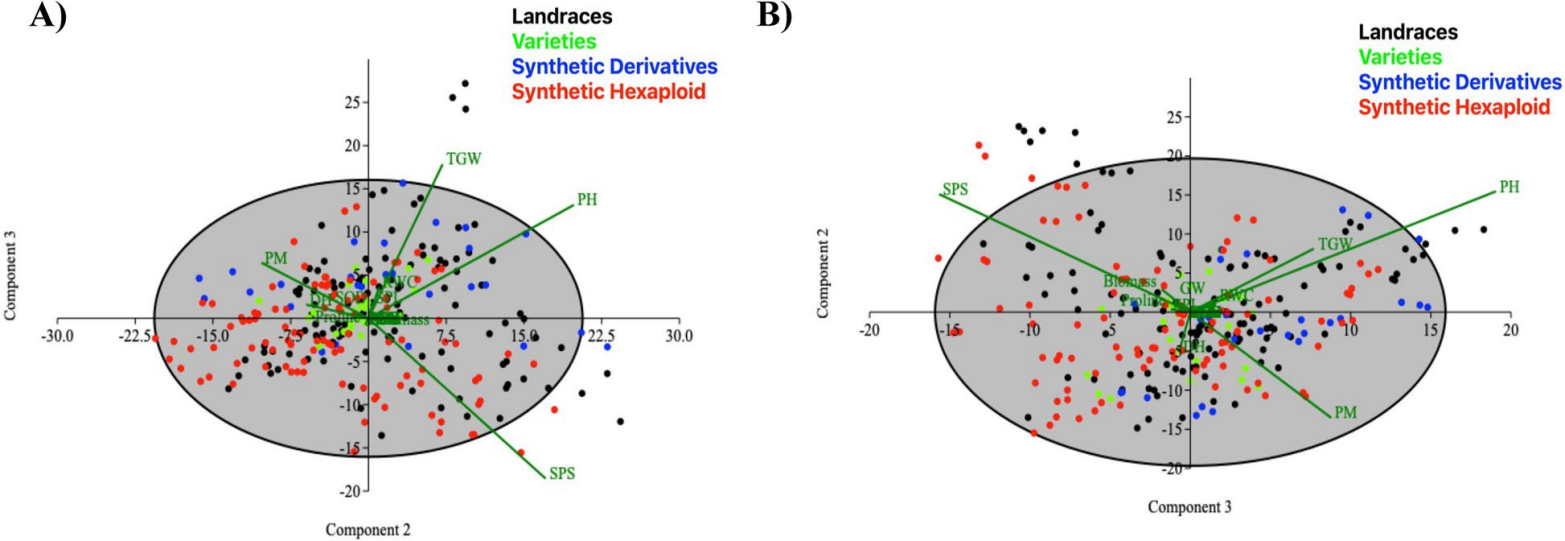
Principal component analysis based biplot showing phenotypes (as vectors) and wheat accessions (as cases) grouped into four different genetic backgrounds under 50% field capacity (**A**), and 30% field capacity (**B**) for the studied phenological and biochemical traits

Principal component analysis (PCA) was carried out for appropriate grouping of the studied traits in response to drought tolerance at 50% and 30% FC in experimental wheats (Fig. 1A and B, ESM 4). The PCA result showed up to 58.63% variation by first two axis i.e. PC1 (eigenvalue = 5.76) and PC2 (eigen value = 3.62). In the first principal component, the contributing traits were DH, PM, PH, SPS, GW, TGW, Biomass, Chla, Chlb and TChl.

### Coefficient of correlation between phenotypic traits under different field capacities

Pearson’s coefficient of correlation revealed positive significant correlation between biomass and GW (*r* = 0.78) and TGW (*r* = 0.33), while it increased to *r* = 0.87 and 0.56, respectively at 30% FC (Tables 2 and 3). At 50% FC, RWC had highest positive correlation with TGW (*r* = 0.89) and GW (*r* = 0.82) followed by biomass (*r* = 0.40), whereas the correlation of RWC with TGW, GW reduced to *r* = 0.72 and 0.86, respectively, in 30% FC. In 30% FC, total chlorophyll showed negative correlation with RWC (*r* = -0.26).

**Table 2:** Coefficient of correlation among phenological and physio-biochemical traits of the studied diverse wheats under control and 30% field capacity

Traits	DH	PM	PH	SPL	SPS	GW	TGW	Biomass	HI	RWC	Proline	Chla)	Chlb	TChl	POD	SOD
DH	—	0.753***	0.044	-0.505***	0.579***	0.527***	0.218***	0.51***	-0.142*	-0.1	0.044	-0.07	-0.112	-0.134	-0.11	0.313**
PM	0.731***	—	0.05	-0.634***	0.599***	0.605***	0.386***	0.597***	-0.204***	-0.116	-0.338**	-0.137	0.151	0.078	-0.155	-0.094
PH	-0.257***	-0.371***	—	-0.117	0.197**	0.247***	0.266***	0.207***	-0.03	0.234*	-0.001	-0.131	-0.032	-0.088	0.007	0.042
SPL	-0.168**	-0.097	0.436***	—	-0.434***	-0.414***	-0.245***	-0.453***	0.258***	0.126	0.052	-0.012	-0.043	-0.045	-0.031	-0.363**
SPS	0.25***	0.413***	0.094	0.369***	—	0.906***	0.437***	0.869***	-0.187**	-0.152	-0.056	0.141	0.144	0.195	-0.194	-0.055
GW	0.253***	0.445***	0.095	0.358***	0.79***	—	0.764***	0.876***	-0.156**	0.723***	-0.118	-0.113	-0.069	-0.113	-0.043	-0.034
TGW	0.096	0.216***	0.088	0.16**	0.107	0.688***	—	0.561***	-0.066	0.862***	-0.127	-0.17	-0.118	-0.184	0.025	-0.03
Biomass	0.335***	0.517***	0.173**	0.386***	0.798***	0.778***	0.335***	—	-0.529***	0.414***	-0.092	0.08	-0.215	-0.161	0.044	0.078
HI	-0.235***	-0.188**	-0.22***	-0.132*	-0.189**	0.047	0.265***	-0.545***	—	0.649***	-0.103	-0.126	-0.028	-0.081	-0.055	-0.053
RWC	0.074	-0.127	0.287*	0.257*	-0.071	0.847***	0.871***	0.483***	0.702***	—	-0.105	-0.204	-0.184	-0.259*	0.135	-0.002
Proline	0.023	-0.088	-0.09	0.029	-0.057	-0.246*	-0.244*	-0.264*	-0.167	-0.351**	—	0.038	-0.04	-0.02***	0.208	0.067
Chla	-0.002	-0.317**	0.105	-0.042	0.036	-0.174	-0.177	0.036	-0.184	-0.17	0.073	—	-0.046	0.402***	-0.075	-0.081
Chlb	0.093	-0.092	-0.245*	-0.242*	-0.076	-0.193	-0.187	-0.052	-0.178	-0.126	-0.046	0.136	—	0.896***	-0.06	-0.039
TChl	0.075	-0.227*	-0.149	-0.217	-0.044	-0.241*	-0.237*	-0.025	-0.233*	-0.184	-0.002	0.592***	0.879***	—	-0.089	-0.072
POD	0.207	-0.02	-0.161	0.039	0.031	-0.166	-0.172	-0.006	-0.159	-0.125	0.176	-0.158	-0.059	-0.124	—	0.073
SOD	0.017	0.255*	-0.082	-0.048	0.023	0.509***	0.512***	0.012	0.5***	0.339**	-0.301**	-0.146	0	-0.07	-0.275*	—

*DH* days to headings, *PM* physiological maturity, *PH* plant height, *SPL* spike length, *SPS* seeds per spike, *GW* grain weight, *TGW* thousand grain weight, *HI* harvest index, *RWC* relative water content, *Chl* chlorophyll, *TChl* total chlorophyll, *POD* peroxidase, *SOD* superoxide dismutase

*, ** and *** are showing statistical significance at the *p*≤0.05, 0.01 and 0.001 probability level

**Table 3:** Coefficient of correlation among phenological and physio-biochemical traits of the studied wheat genotypes under control and 50% field capacity

Traits	DH	PM	PH	SPL	SPS	GW	TGW	Biomass	HI	RWC	Proline	Chla	Chlb	TChl	POD	SOD
DH	—	0.598***	-0.09	-0.28***	0.303***	0.354***	0.216***	0.342***	-0.098	0.075	0.162	-0.252*	-0.126	-0.203	-0.069	0.004
PM	0.731***	—	-0.057	-0.354***	0.524***	0.559***	0.308***	0.679***	-0.319***	-0.115	-0.044	-0.309**	0.025	-0.084	-0.076	0.134
PH	-0.257***	-0.371***	—	0.399***	0.217***	0.277***	0.279***	0.268***	-0.144*	0.235*	-0.121	-0.038	-0.07	-0.077	0.019	-0.027
SPL	-0.168**	-0.097	0.436***	—	0.09	0.136*	0.157**	0.041	0.04	0.27*	0.104	0.153	-0.006	0.047	-0.107	-0.025
SPS	0.25***	0.413***	0.094	0.369***	—	0.853***	0.243***	0.788***	-0.096	-0.167	0.093	0.16	-0.046	0.013	-0.034	0.102
GW	0.253***	0.445***	0.095	0.358***	0.79***	—	0.706***	0.831***	-0.109	0.819***	-0.077	-0.209	-0.113	-0.177	0.109	0.375***
TGW	0.096	0.216***	0.088	0.16**	0.107	0.688***	—	0.486***	-0.087	0.893***	-0.099	-0.25*	-0.104	-0.182	0.122	0.357**
Biomass	0.335***	0.517***	0.173**	0.386***	0.798***	0.778***	0.335***	—	-0.586***	0.398***	-0.225*	0.008	-0.174	-0.158	0.146	0.357**
HI	-0.235***	-0.188**	-0.22***	-0.132*	-0.189**	0.047	0.265***	-0.545***	—	0.726***	-0.018	-0.21	-0.074	-0.141	0.078	0.296**
RWC	0.074	-0.127	0.287*	0.257*	-0.071	0.847***	0.871***	0.483***	0.702***	—	-0.204	-0.278*	-0.131	-0.217	0.124	0.382***
Proline	0.023	-0.088	-0.09	0.029	-0.057	-0.246*	-0.244*	-0.264*	-0.167	-0.351**	—	0.141	0.235*	0.266*	0.075	-0.017
Chla	-0.002	-0.317**	0.105	-0.042	0.036	-0.174	-0.177	0.036	-0.184	-0.17	0.073	—	0.043	0.386***	-0.111	-0.092
Chlb	0.093	-0.092	-0.245*	-0.242*	-0.076	-0.193	-0.187	-0.052	-0.178	-0.126	-0.046	0.136	—	0.938***	-0.149	-0.203
TCh	0.075	-0.227*	-0.149	-0.217	-0.044	-0.241*	-0.237*	-0.025	-0.233*	-0.184	-0.002	0.592***	0.879***	—	-0.176	-0.219
POD	0.207	-0.02	-0.161	0.039	0.031	-0.166	-0.172	-0.006	-0.159	-0.125	0.176	-0.158	-0.059	-0.124	—	0.285*
SOD	0.017	0.255*	-0.082	-0.048	0.023	0.509***	0.512***	0.012	0.5***	0.339**	-0.301**	-0.146	0	-0.07	-0.275*	—

*DH* days to headings, *PM* physiological maturity, *PH* plant height, *SPL* spike length, *SPS* seeds per spike, *GW* grain weight, *TGW* thousand grain weight, *HI* harvest index, *RWC* relative water content, *Chl* chlorophyll, *TChl* total chlorophyll, *POD* peroxidase, *SOD* superoxide dismutase

*, ** and *** are showing statistical significance at the *p*≤0.05, 0.01 and 0.001 probability level

Drought stress tolerance index (STI) which takes into account, the yield under stress conditions compared to yield potential of a cultivar, classified the studied wheats as stress tolerant (STI > 1.0), moderately tolerant (STI between 0.5 to 1.0), or stress susceptible (STI ≤ 0.5). The studied wheats which exhibited STI above 1 both under 30 and 50% FC included AA-51, Pak-13, LR-87A, LR-77, NARC-09, SD-212, SD-177, LR-37, LR-61, LR-53B, SD-227, LR-75, LR-7, LR-39, SH-DArT-119, LR-40, SH-DArT-2, LR-36 and SH-DArT-3, which represented landraces, cultivars/varieties and synthetic derived wheats (ESM 6).

Regarding genotypes overall performance, an ordinary trait scoring was done both under 50% and 30% FC environment, for the studied traits. The top 20% genotypes were assigned score 3, the next 20% with score 2 and the rest with score 1 (ESM 7 and 8). Under 50% FC, the highest score (24) was recorded for LR-09, LR-42 and LR-51; followed by LR-18 (23), LR-10 (22), LR-50 (22), Krichauf (22), LR-19B (21), SD-212 (21), SD-177 (21) and NARC-09 (21). Similarly, under severe stress condition, the maximum score (25) was recorded for LR-13, followed by LR-10 (23), LR-18 (23), LR-42 (23), LR-51 (22), SD-177 (22), SD-212 (21), LR-9 (21), NARC-09 (20), FSD-08 (20), LR-40 (19) and Chirya-1 (19). Conclusively, the landraces were much promising along with varieties and synthetic derived wheats.

### Allelic variations and effects of drought tolerance related genes

KASP genotyping was carried out and allelic frequency of the genes was calculated (Table 4). For *TaSnRK2.9-5A-KASP-5*, *Hap-*CA was identified in 16 genotypes (17.6%) whereas *Hap*-CC was identified in 45 accessions (49.5%). The *Hap-*TA and *Hap-*TC of *TaSnRK2.9-5A-KASP-6* were recognized in 19 (20.9%) and 11 genotypes (12.1%), respectively. The *Hap*-A was identified in 43 (53.1%) and *Hap*-G was present in 38 accessions (46.9%) for *TaLTP-KASP-11,* whereas in *TaLTP-KASP-12 Hap*-C was identified in 63 accessions (70.8%) and *Hap*-T was identified in 26 (29.2%) accessions. The Kauz-type allele was identified in 37 (47.4%), while Westonia-type allele was identified in 41 accessions (52.6%). In *Dreb-B1 Hap*-A was recorded in 45 (57.7%) and *Hap*-C was identified in 33 genotypes (42.3%).

**Table 4:** Allelic frequencies of the studied diverse wheats for drought related genes

Gene	Primer Name	Allele	Number of genotypes	Frequency
*TaSnRK2.9-*5A	*TaSnRK2.9-5A-KASP-5*	Hap-CA	16	17.6%
		Hap-CC	45	49.5%
	*TaSnRK2.9-5A-KASP-6*	Hap-TA	19	20.9%
		Hap-TC	11	12.1%
*TaLTPs*	*TaLTPs-KASP-11*	Hap-A	43	53.1%
		Hap-G	38	46.9%
	*TaLTPs-KASP-12*	Hap-C	63	70.8%
		Hap-T	26	29.2%
*1feh-w3*	*1fehw3*	Kauz type	37	47.4%
		Westonia type	41	52.6%
*Dreb*	*Dreb-B1*	Hap-A	45	57.7%
		Hap-C	33	42.3%
*TaPPH-*7A	*TaPPH-KASP-13*	Hap-A	42	47.2%
		Hap-G	47	52.8%
*TaSAP-*7B	*TaSAP-7B-KASP-8*	Hap-C	66	76.7%
		Hap-T	20	23.3%

**Fig. 2:**
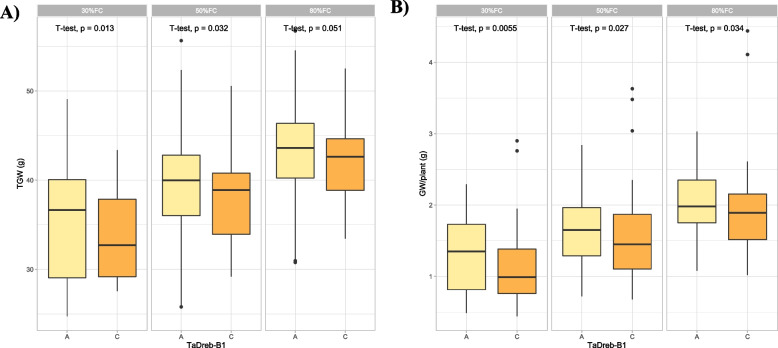
Box plots showing allelic effects of *TaDreb-B1* gene on thousand grain weight (**A**) and grain weight per plant (**B**) under three different field capacities i.e. 30%, 50% and 80%. The effects of two alleles identified in *TaDreb-B1* (A versus C) were statistically compared using student’s t-test and *p*-values are shown in each water treatment

**Fig. 3:**
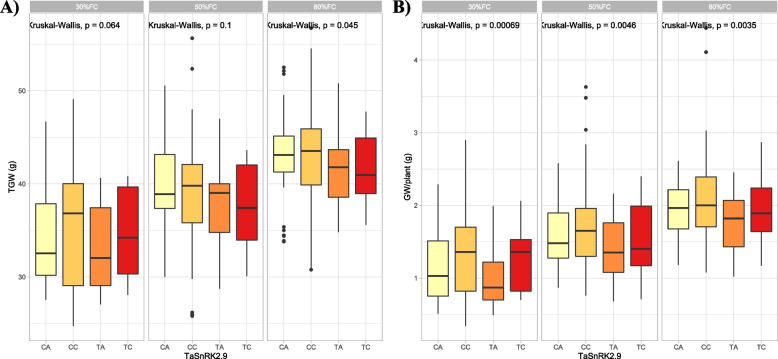
Box plots showing allelic effects of *TaSnRK2.9-5A* gene on thousand grain weight (**A**) and grain weight per plant (**B**) under three different field capacities i.e. 30%, 50% and 80%. The effects of four alleles identified in *TaSnRK2.9* (CA, CC, TA, and TC) were statistically compared using Kruskal–Wallis test and *p*-values are shown in each water treatment

**Fig. 4:**
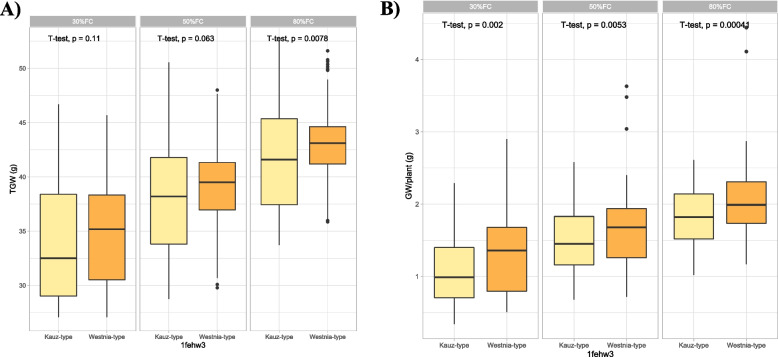
Box plots showing allelic effects of *1fehw3* gene on thousand grain weight (**A**) and grain weight per plant (**B**) under three different field capacities i.e. 30%, 50% and 80%. The effects of two alleles identified in *1fehw3* (Kauz-type versus Westonia-type) were statistically compared using student’s t-test and *p*-values are shown in each water treatment

The allelic effects of each gene on each phenotype are presented in ESM 5. Allelic variation in *TaDreb-B1* showed significant effects on TGW at both 50% and 30% FCs, whereas GW exhibited difference at 80% and 50% FCs (Fig. 2). Likewise, four different haplotypes of *TaSnRK2.9-5A* were also associated with GW across three different FCs, while TGW was observed significant at 80% FC (Fig. 3). The favourable haplotype “CC” increased GW and was present in 49.5% accessions. The Westonia-type allele was observed in accessions with higher GW, TGW and biomass, whereas, the Kauz-type allele slightly reduced the GW, TGW and biomass under water deficit stress. Allelic variation in *1fehw3* showed substantial effect on GW across three FCs while no significant effect on TGW under drought stress (Fig. 4).

**Fig. 5:**
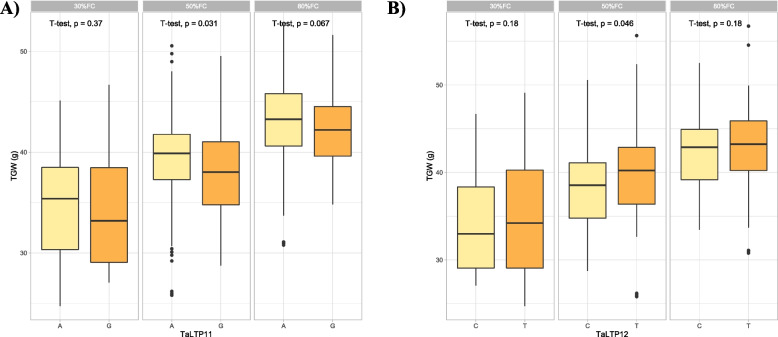
Box plots showing allelic effects of *TaLTP-KASP-11* (A) and *TaTLP-KASP-12* (B)on thousand grain weight under three different field capacities i.e. 30%, 50% and 80%. The effects of two alleles identified in both genes were statistically compared using student’s t-test and *p*-values are shown in each water treatment

Under drought stress, alleles of *TaLTP-KASP-11* and *TaLTP-KASP-12* were also associated with GW and TGW (Fig. 5). The allelic frequency of the favourable alleles was almost similar for both genes.

## Discussion

### Comparison of the germplasm groups under water-limited conditions

Development of drought tolerant wheat varieties is one of the most important breeding objective. Screening diverse wheat genotypes under water limited conditions is considered an efficient means of selecting germplasm for advanced breeding programs [43, 75, 76]. In current study, All the wheat germplasm showed variation in their responses under control and progressive drought stress condition. Landraces showed greater variation for morphological and biochemical traits compared to synthetic hexaploids and improved cultivars. However, least reduction in grain weight was observed in improved cultivars which indicated the progress in developing drought resilient cultivars. The reduction in heading time under drought stress may reflect positive response in several bread and durum wheat varieties [77], however it does not ensure higher yield [12, 78, 79]. Seher et al. [80] evaluated a collection of landraces and synthetic derivatives and reported that the later has higher TGW as compared to landraces. The current study agreed with the results of Baser et al. [81] who reported significant reduced GW and TGW under drought stress. Placido et al. [82] reported that the landraces were least affected by drought stress as compared to other wheat germplasm. Various studies revealed that synthetic derivatives produce 23% higher yield than local cultivars under drought stress [24]. Such studies are important to identify the germplasm resources with least effect on yield related phenotypes under drought stress. Our results iterated the fact that landraces could be promising source to deploy drought adaptability in wheat breeding. On the whole, these results as well as those from others suggest that selecting strategy would be reliable if based on early flowering, grain number per spike, grain yield per plant and most importantly upon STI for increasing yields under drought conditions [61, 62]. Accordingly, among the studied genotypes, SD-212, SD-177 and LR-40, which exhibited STI greater than 1, also performed well in arbitrary scoring (ESM 7 and 8), and were therefore recommended for further micro-yield wheat trials.

### Allelic effects on functional genes on drought adaptability

This use of molecular markers for selecting genotypes with favourable alleles of major genes offers an opportunity to efficiently select and use genetic resources in wheat breeding. Marker-assisted selection of favourable alleles in breeding programs is essential in wheat development. Accessibility of resourceful molecular knowledge may lead to enhanced applicability of superior alleles in cultivars overall improvement [83–86]. Further, genetic studies carried out through KASP assays have made it feasible to genotype diverse population at different loci in limited time [87]. Number of recent research work have utilized KASP markers for exploring the allelic diversity of functional genes in wheat cultivars from the United States [88]; China [87] and Canada [89]. Genes studied in this work and their allelic variation were found associated with morphological characteristics under control and drought stress condition. The current findings are in agreement with the study of Rehman et al. [51] who observed significant association of *TaSnRK2.9-5A* with TGS and SPS.

*Dreb1* genes are positioned on chromosome 3A, 3B and 3D in wheat genome. Genetic mapping of *Dreb-B1* genes by Gao et al. [90] revealed that it is positioned on 3BL chromosome amongst *Xmwg818* and *Xfbb117*. Wei et al. [50] stated that *Dreb1* genes are associated with environmental-stress tolerance like temperature, salinity, ABA and mostly drought. In wheat, the *Dreb-B1* gene provides resistance against drought stress. Zhang et al. [91] reported that alleles of *1fehw3* significantly associated with higher TGW under drought stress. The current research work is in line with that of Wang et al. [54] who reported significant association of *TaPPH-7A* with TGW and chlorophyll (b) content. Wang et al., [92] revealed that stress associated proteins (SAPs) are the A20/A1 zinc-finger proteins negotiating environmental stress in plants. *TaSAP-7B* possess two alleles or haplotypes *Hap-*C and *Hap-T* which are associated with TGW and PH, whereas the current study also demonstrated the same result. The present work is in general agreement with that of Wang et al. [92] who reported that the germplasm acquiring *Hap-*C show higher TGW and smaller PH.

Marker assisted selection focusing superior alleles is considered essential for wheat improvement in ongoing breeding programs. Further, deployment/utilization of superior alleles could be improved subject to availability of efficient molecular diagnostics. Previously, promising allelic variations of the genes have been reported to be associated with higher grain weight and higher grain number under control and drought stress conditions [84, 85]. Current research may have positively impacted the need for assessing the effect of selection pressure on favorable haplotypes and to alert wheat breeders for favorable variations for grain yield. Previously, moderate frequency of favored haplotypes was being observed at *TaDreb*-B1, *TaSnRK2.8*-5A, *1-feh w3*, *TaPPH*-7A, which further indicated that exploitation of these alleles may be continued for attaining enhanced gain yield. The resulting unconscious selection of favorable haplotypes may be attributed to high linkage disequilibrium of important genes selected during selection breeding. [51, 70, 81]. favorable allelic variations in Chinese wheat cultivars where the frequencies of favored haplotypes had gradually increased from the beginning of the last century. Hence, introgression from different wheats may be a preferred strategy to introduce novel allelic variations at loci conferring drought tolerance for sustainable production.

## Conclusion

Prolonged drought stress affects the metabolic reaction of plants associated with growth and yield characteristics. Improvement in yield under drought stress has been identified as a tough challenge for plant breeders. In this study thousand grain weight (TGW) and harvest index (HI) were least affected by applied drought regimes. Among the investigated molecular markers, *Dreb-B1*and *TaPPH-7A-KASP-13* showed highest association for drought tolerance. Because of constantly changing environment an allelic combination may be needed for the adaptation of wheat to drought stress. Further, with new breeding approaches, it is essential to screen the effect of drought stress and genes that continuously influence it. The findings will be helpful to identify genotypes with water-saving alleles and will provide an insight knowledge that could assist wheat breeders in introgression /combining of favorable genes into new cultivars through marker assisted selection.

## Supplementary Information

**Additional file 1: ESM 1.** Pedigree of the experimental wheat germplasm. **ESM 2.** List of KASP primers and their sequences used during this study. **ESM 3.** Analysis of variance for the studied wheat genotypes. **ESM 4.** Principal componenet analysis (PCA) of the studied traits in expermental genotypes. **ESM 5.** Allelic effects of the studied wheat genotypes under drought stress. **ESM 6.** Ranking of the studied wheats based on the stress tolerance index. **ESM 6.** Arbitrary scoring of the genotypes for the studied wheat genotypes under 50%. For each trait, top 20 % genotypes were assigned score 3, Next 20 % genotypes with score 2 and the lowest with score 1. **ESM 7.** Arbitrary scoring of the genotypes for the studied wheat genotypes under 30% FC. For each trait, top 20 % genotypes were assigned score 3, Next 20 % genotypes with score 2 and the lowest with score 1.

## Data Availability

The phenotypic and genotypic data is available in the supplementary data files.
